# PA OmniNet: A retraining-free, generalizable deep learning framework for robust photoacoustic image reconstruction

**DOI:** 10.1016/j.pacs.2025.100740

**Published:** 2025-07-08

**Authors:** Olivier J.M. Stam, Kalloor Joseph Francis, Navchetan Awasthi

**Affiliations:** a Faculty of Science, Mathematics and Computer Science, Informatics Institute, University of Amsterdam, Amsterdam, 1090 GH, The Netherlands; b Erasmus MC, Cardiovascular Institute, Department of Cardiology, Biomedical Engineering, Rotterdam, The Netherlands; c Department of Biomedical Engineering and Physics, Amsterdam UMC, Amsterdam, 1081 HV, The Netherlands

**Keywords:** Photoacoustic imaging, Sparse sampling, Deep learning, U-net, Retraining-free, Generalizable, Reconstruction, Few-shot learning

## Abstract

For clinical translation of photoacoustic imaging cost-effective systems development is necessary. One approach is the use of fewer transducer elements and acquisition channels combined with sparse sampling. However, this approach introduces reconstruction artifacts that degrade image quality. While deep learning models such as U-net have shown promise in reconstructing images from limited data, they typically require retraining for each new system configuration, a process that demands more data and increased computational resources. In this work, we introduce PA OmniNet, a modified U-net model designed to generalize across different system configurations without the need for retraining. Instead of retraining, PA OmniNet adapts to a new system using only a small set of example images (between 4 and 32), known as a context set. This context set conditions the model to effectively remove artifacts from new input images in various sparse sampling photoacoustic imaging applications. We evaluated PA OmniNet against a standard U-net using multiple datasets, including in vivo data from mouse and human subjects, synthetic data, and images captured at different wavelengths. PA OmniNet consistently outperformed the traditional U-net in generalization tasks, achieving average improvements of 8.3% in the Structural Similarity Index, a 11.6% reduction in Root Mean Square Error, and a 1.55 dB increase in Peak Signal-to-Noise Ratio. In 66% of our test cases, the generalized PA OmniNet even outperformed U-net models trained specifically on the new dataset. Code is available at https://github.com/olivierstam4/PA_OmniNet.

## 1 Introduction

Photoacoustic imaging (PAI) has received increasing attention in the past two decades due to its capability of combining optical contrast with acoustic resolution and depth. [1], [2]. In photoacoustic imaging nanosecond pulsed illumination induces acoustic signal from tissue absorbers due to thermoelastic expansion. [3], [4], [5]. PAI is a promising modality for diverse biomedical applications, including cardiovascular imaging and cancer imaging. [4], [6], [7], [8].

Despite its potential, widespread adoption of PAI is hindered by high costs [9] and slow imaging speeds [10]. An ideal image requires collecting photoacoustic signals in all directions with an infinite number of detectors. In a practical case, the view angle and the number of transducer elements available are limited. A higher number of transducer and acquisition channels increases the acquisition and processing time as well as the system cost [9]. To address these limitations, sparse sampling techniques have been proposed, which reduce the number of transducers required [11]. However, this approach introduces streak-type artifacts in the reconstructed image that degrade image quality. Despite these limitations, great strides have been made in addressing these issues, particularly through advances in deep learning and reconstruction algorithms.

Recent advances in deep learning have shown the potential to mitigate these issues. Convolutional neural networks (CNN), particularly U-Net-based architectures, have shown success in reducing artifacts and improving image reconstruction quality. Using these models, PAI can be performed more cost-effectively and efficiently. Deep learning architectures, such as U-net and its variants, have shown remarkable success in reconstructing sparse sampled PAI data [12]. Although originally designed for image segmentation, U-nets can easily be adapted to work on image reconstruction tasks.

In a 2019 study, Davoudi et al. [11], developed a CNN trained on sparse sampled photoacoustic data that effectively reduces streak artifacts and improves visibility of anatomical characteristics. Their model, a U-net-like architecture, was trained on high-quality in vivo data from mouse, representing characteristics of real-world conditions. Building on this U-net-based framework, they introduced HD-U-net to enhance PAI frame rates by reconstructing high-quality images from sparse signal acquisition was proposed [10]. Their model incorporates dilated dense blocks and demonstrates significant improvements in imaging speeds. Although this work shows a step forward in imaging speeds, the model remains reliant on task-specific training data, limiting implementation due to variations in real-world scenarios. ModUnet, a modified U-net designed for sparse sampling, limited-view artifacts, and anatomical segmentation is another related development [13]. While effective, this approach requires a total of 44 pre-trained models, each specialized for a specific, single task, highlighting the computational inefficiency of creating task-specific models. Models trained on specific datasets often fail to perform well on new applications without retraining or fine-tuning, which necessitates large datasets and significant computing power. Moreover, existing datasets usually lack diversity as they often focus on a single subject, further limiting the generalization of trained networks. Even though U-nets excel in specific use cases [14], they are known to have poor generalizability [15]. Training multiple specialized U-nets for different applications is not only computationally expensive but also necessitates datasets with labels and ground truths, which are often scarce in photoacoustic imaging. In addition, medical personnel often lack the time or knowledge to fine-tune these models for specific tasks. This raises a critical question: Can a generalized deep learning model be developed that effectively removes artifacts and enhances image reconstruction quality across different sparse sampling (limited data and limited view) PAI applications without the need for retraining?

In this work, we introduce PA OmniNet, a deep-learning model for artifact removal and high-resolution image reconstruction in sparsely sampled photoacoustic imaging (PAI). Inspired by the Neuralizer architecture, which has demonstrated success in MRI image processing [16], PA OmniNet adapts to new photoacoustic systems using a context set of 4 – 32 labeled example images, enhancing generalizability. Unlike traditional models, PA OmniNet generalizes in a single forward pass, eliminating the need for retraining or fine-tuning. This approach not only reduces computational costs, requiring only a single trained model, but also ensures robust performance across diverse datasets and imaging systems. We have evaluated PA OmniNet on multiple datasets from different system configurations, compared it to a U-Net based reconstruction and also tested its generalization capability. Our results demonstrate that PA OmniNet effectively removes artifacts across varied sparse-sampling PAI applications, both quantitatively and visually. By addressing key generalization challenges, this work represents a step toward the broader clinical adoption of deep learning in PAI. See Table 1 for abbreviations used in this work.

**Table 1:** Abbreviations of all the terms used..

Abbreviation	Full form
PAI	Photoacoustic imaging
CNN	Convolutional neural network
PAT	Photoacoustic tomography
PSNR	Peak signal to noise ratio
SSIM	Structural similarity index metric
MSE	Root mean square error
RMSE	Root mean square error
SRMSE	Scaled root-mean-square error
Alpha	Combination loss function of MSE and SSIM
ELUs	Exponential linear units
MSFD	Multispectral forearm dataset
SWFD	Single wavelength forearm dataset
SCD	Simulated cylinders dataset

## 2 Methods

### 2.1 Proposed model: PA OmniNet

**Fig. 1:**
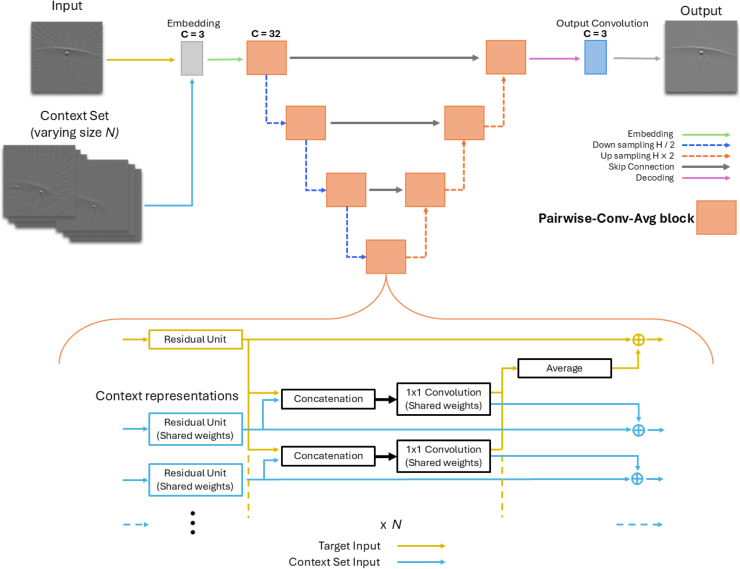
PA OmniNet architecture with U-net-like structure with Pairwise-Conv-Avg blocks (top) and a detailed view of the Pairwise-Conv-Avg block (bottom).

In this section we present the architecture of the proposed PA OmniNet, an adaptation of the Neuralizer model by Czolbe et al. [16]. Built upon a U-Net architecture, PA OmniNet distinguishes itself by leveraging a small set of labeled input–output examples (4 to 32), referred to as the context set, to generalize to new imaging systems in a single forward pass, without retraining or fine-tuning. This adaptation is enabled by Pairwise-Conv-Avg blocks (Fig. 1). These blocks facilitate learned weight transfer from the context set to the model by combining pairwise convolutions with averaging operations. The PA OmniNet architecture, as illustrated in Fig. 1, consists of an embedding layer, seven Pairwise-Conv-Avg blocks arranged in a U-net-like configuration and an output convolution layer.

#### 2.1.1 Context set

The context set is a small set of labeled input–output images, usually ranging from 4–32 images, and is needed to complete the forward pass. Both the context-in and context-out are given as separate inputs to the model. The model relies on the context set to generalize, however due to the robustness of the model, it is not necessary to take the context set of that specific application during the inference process. The model will output satisfactory images as long as the context set is an approximate representation of the task. During the training phase it is recommended to use a context set from the training data.

#### 2.1.2 Pairwise-Conv-Avg block

In a Pairwise-Conv-Avg block, the input image and the context set go into the block as three inputs and are passed through residual units, which apply two convolutions. The residual units that process the context-in and context-out have shared weights. As can be seen in Fig. 1 (bottom), for every individual pair of the context set the black blocks are repeated. Therefore the context set does not go through the singular residual unit as a whole, but is split up in pairs for the different context inputs. This process allows every input image to interact with the entire context set while each context-pair can interact and share information with the target input. Next, the context is pairwise concatenated with the target representation on the channel dimension. After that, a 1 × 1 convolution is applied to reduce the channel size back to the original channel dimension and the target representation is updated by averaging across context members. Finally, the output is resized before being fed to the next block (Fig. 1 (top)) [16]. The use of these Pairwise-Conv-Avg blocks ensures that all the information across the context set is effectively used, enabling robust generalization. Because of the averaging function in the Pairwise-Conv-Avg block, the model remains invariant to context size change, enabling flexible generalization across tasks. This flexibility is crucial for real-world PAI applications, where the availability of labeled data may vary. Using this design, PA OmniNet can adapt to new applications simply by passing a related context set. While training, it is important to keep the context set size low. In this way, the model learns not to depend only on the context set but can also extract more features from the context set in different testing situations, while at the same time learning how to incorporate the given context.

### 2.2 U-net

To benchmark the performance and generalization capabilities of the proposed model, it is essential to compare it against a state-of-the-art U-net model. Since the proposed model, PA OmniNet, is derived from a U-net framework, selecting a high-performance benchmark model is crucial for a meaningful comparison. Therefore, the hybrid U-net model has been chosen, as it is designed to tackle challenges in photoacoustic tomography (PAT) and has demonstrated excellent performance on reconstruction with sparse sampled data [14]. The hybrid U-net model effectively handles bandwidth limitations, noise reduction, and sparse data [14]. This model is particularly effective, as it processes the photoacoustic image as a whole, rather than focusing on individual pixels, enhancing robustness and reducing streak artifacts. To address the challenges posed by extremely low magnitude values within the image, the model introduces a scaled root-mean-square error (SRMSE) loss function. This loss function ensures stable training by scaling the gradients, preventing the vanishing gradient problem. Additionally, the model incorporates exponential linear units (ELUs) in critical layers, which are better suited for handling negative values compared to traditional activation functions like ReLU. The hybrid U-net’s ability to deliver high-quality reconstructions under sparse sampled data makes it an ideal comparison model.

### 2.3 Datasets

To evaluate the generalization of the proposed model, we test it across a variety of datasets representing different sparse sampling applications of PAI. By leveraging multiple datasets, the model’s ability to generalize across dissimilar sparse sampling applications can be properly assessed. This approach ensures that the model’s performance is not limited to a single dataset or application, but can be reliably applied to a broad range of photoacoustic sparse sampling applications. In this section, the datasets used in this work are described, along with their importance, uniqueness, and specific use case.

#### 2.3.1 OADAT

**Fig. 2:**
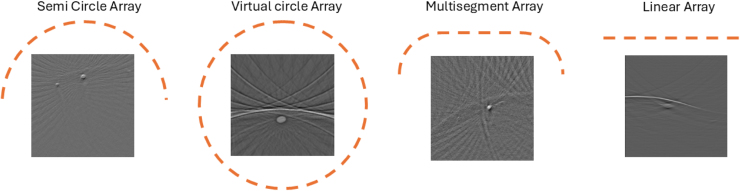
Transducer array configurations used in the OADAT sub-datasets.

The OADAT dataset, introduced by Ozdemir et al. [13], consists of four sub-datasets. For this work, three sub-datasets are used, as one is scaled down for simplicity. Although the dataset provides both raw photoacoustic data and preprocessed image data, this work will focus exclusively on preprocessed image data, as the primary objective of the proposed model is to improve the reconstruction of sparse data into high-resolution images. The sub-datasets contain sparse sampling conditions ranging from 32 to 128 transducers and fully sampled ground truth images (Table 2). The various sub-datasets are explained in the sub-sections below:

1. **Multispectral Forearm Dataset (MSFD)** The MSFD comprises real-world data collected from the forearms of nine volunteers using a multi-segment array across six different wavelengths (700–850 nm) (Table 2). It contains raw data for linear and multi-segment arrays (Fig. 2), but it only features preprocessed images for the multi-segment arrays. Therefore, linear arrays have not been used in experiments. This sub-dataset is well suited to study wavelength-dependent phenomena, such as the properties of different tissues. In this work, the 780 nm wavelength is used, with the input from a multi-segment transducer 32 sparse sampled, and the ground truth is multi-segment, fully sampled. The ground truth is generated using a 256-transducer setup, with 128 transducers in the linear section and 64 transducers on each concave section. By using this sub-dataset, the model can be tested on image reconstruction for sparse sampling using a different wavelength.
2. **Single Wavelength Forearm Dataset (SWFD)** The SWFD sub-dataset simplifies the images by collecting data at a singular wavelength (1064 nm) from the forearms of 14 volunteers (Table 2). This dataset contains sparse sampling from semicircle and multi-segment arrays (Fig. 2), splitting the dataset into SWFD Semi and SWFD Multi, respectively. SWFD Semi takes semi-circle, 32 sparse sampled as input and semi-circle, fully sampled as the ground truth, generated using 256 transducers. Similarly, for SWFD Multi, the input is multi-segment, 32 sparse sampled and the ground truth is multi-segment, fully sampled, using the same configuration and transducer count as the MSFD sub-dataset. This subset directly compares the model’s performance across different transducer geometries, testing how it would perform on varying real-world hardware configurations. The SWFD Semi sub-dataset will be used to train the proposed model for generalization.
3. **Simulated Cylinders Dataset (SCD)** Lastly, the SCD is a synthetically generated dataset designed to bridge the gap between real-world data and controlled experiments by simulating forearm acoustic pressure maps and artifacts (Table 2). This makes it possible to evaluate the model on idealized but challenging test objects. The input is multi-segment, 32 sparse sampled and the ground truth is multi-segment, fully sampled. The fully sampled data is gathered by a simulated multi-segment array with 256 transducers. By including synthetic data, the model’s performance can be tested in a controlled environment.

**Table 2:** Details of the used OADAT sub-datasets in the study. Each sub-dataset includes sparse sampling configurations with 32 transducers (input) and fully sampled ground truth images (output). The table specifies the training, validation, and testing splits for each sub-dataset, along with the context set used for testing.

Dataset	Train	Validation	Test	Size	Input (key)	Output (key)	Testing context neuralizer
MSFD	patientID [2,5,6,7,9]	patientID [10,11]	patientID [14,15]	14000/5600/5600	32 sparse sampled	Ground truth	First 16 context pairs patientID 2
SWFD Semi	patientID [1-10]	patientID [11,12]	patientID [13,14]	28020/5600/5600	32 sparse sampled	Ground truth	First 16 context pairs patientID 5
SWFD Multi	patientID [1-10]	patientID [11,12]	patientID [13,14]	28020/5600/5600	32 sparse sampled	Ground truth	First 16 context pairs patientID 5
SCD	70%	20%	10%	14000/4000/2000	32 sparse sampled	Ground truth	First 16 context pairs training data

#### 2.3.2 Mouse dataset

The mouse dataset provides a combination of real-world and controlled experimental data [11]. This dataset is divided into two sub-datasets, each designed to address different aspects of PAI: In vivo mouse data and vascular phantom data (Table 3). Together, they provide a combination of real-world and controlled PAI conditions. Sparse sampling configurations range from 4 to 512 transducers, with 512 serving as the ground truth. Transducer counts ranging from 16 to 128 sparse sampling configurations have been used for training and testing to evaluate the model’s robustness.

##### In vivo mouse data.

The in vivo dataset collected data from six different mouse positioned in a water tank. Using a ring array, each mouse was vertically scanned over 50 mm in 0.5 mm steps, resulting in 100 cross-sectional images that covered the shoulders to the lower abdomen [11]. For these scans, a 1064 nm wavelength was used, the same as in the OADAT SWFD sub-dataset. This sub-dataset is valuable for testing the model’s performance on real-world data and variability (Table 3).

**Table 3:** Details of the three mouse sub-datasets used in the study. Each sub-dataset includes sparse sampling configurations ranging from 4 to 512 transducers with 16/32/64/128 used as input and 512 as ground truth. The table specifies the training, validation, and testing splits for each sub-dataset, along with the context set used for testing.

Dataset	Train	Validation	Test	Size	Input	Output	Testing context PA omninet
In vivo mouse	70%	20%	10%	191/54/29	Mouse 16/32/64/128 sparse	Mouse full recon	First 16 context pairs training data
Vascular phantom	60%	20%	20%	60/20/20	Vphantom 16/32 sparse	Vphantom full recon	First 16 context pairs training data

##### Vascular phantom data.

The vascular phantom (Vphantom) sub-dataset scales down the detail of in vivo data by featuring complex patterns that replicate blood vessels. A total of 33 vascular phantoms were captured, with data augmentation increasing the size to 100 images (Table 3). This sub-dataset is ideal for assessing the model’s ability to handle complex structures and subtle variations.

#### 2.3.3 Model training and testing

We rigorously evaluated the performance and generalization capabilities of the PA OmniNet across multiple datasets, transducer counts and acquisition setups, comparing it to the conventional U-Net as well as its task-specific PA OmniNet models. Our experimental framework was designed to assess not only the model’s ability to perform accurately on diverse datasets but also its robustness in generalizing to dissimilar datasets, to evaluate its overall efficiency.

#### 2.3.4 Training strategy

The training process has been carefully designed to ensure a fair comparison between PA OmniNet and the U-Net model. Early stopping is employed to prevent overfitting, with the best model selected based on its validation loss, while all models are trained on images resized to 256 × 256 pixels to enable a direct comparison between the generalization and task-specific models. Both U-net and PA OmniNet have been trained and validated with different loss functions. These being a standard Mean Square Error (MSE) loss function from the PyTorch Functional library [17], Structural Similarity Index (SSIM) [18], [19], and lastly a combination of the two, called Alpha loss. This loss is calculated according to the following formula:

(1) Loss=(1−α)⋅MSE+α⋅SSIM

This work used an α of 0.84 [20]. The context set for PA OmniNet is generated using a sliding-window approach over the training data with a window size of 4, which prevents the model from overly relying on the context size and thus gaining an advantage during testing, where the context set size is increased to 16. By using the sliding window technique, every input image is accompanied by the next 4 input–output-pairs serving as the context set. This way the model can learn how to extract details out of a different context set for every input during the training phase. These pairs are concatenated to the input in the Pairwise-Conv-Avg block, as explained in Section 2.1.2. For all the models, a standard learning rate of $1e^{-4}$ is enforced in combination with the Adam optimizer from the PyTorch Optimization library [17]. The maximum training epochs was set to 500 for all the models, with all PA OmniNet MSE and Unet models converging far before while PA OmniNet Alpha and SSIM models were stopped around 500 epochs.

#### 2.3.5 Inference process

During the inference process the model handles the data in largely the same manner as in the training phase. Although the context size is increased to 16, every input image is accompanied by the entire context set. This context set is now static, compared to the sliding-window technique of the training phase, the first 16 input–output pairs of the training data are now used as the context set. The input and context set communicate in the same way as in the training phase, as explained in Section 2.1.2.

#### 2.3.6 Testing generalization capability

To evaluate the generalization of our proposed model, we train it on the OADAT SWFD Semi sub-dataset (2.), which provides a solid and sufficiently sized foundation as a base dataset. A U-Net model is also trained on this dataset; these two models are referred to as the Generalized models. In addition, task-specific models are trained on each individual sub-dataset (Section 2.3), these are referred to as the Specific models. This approach allows us to compare both the generalization capabilities and the task-specific accuracy of the models. We also conduct tests on both the OADAT and Mice sub-datasets to evaluate the impact of using a different context set, the effect of varying the context size, and the sensitivity of the models. In total, 60 models are trained and evaluated across the different datasets. For testing, we use three quantitative evaluation metrics to assess various aspects of image reconstruction, complemented by qualitative evaluation through visual inspection.

### 2.4 Evaluation metrics

To evaluate the performance of the models full reference image quality metrics were utilized. To address different aspects such as noise and structural similarity of the output with respect to the ground truth, three distinct quantitative metrics have been selected: Root Mean Square Error (RMSE) [21], [22], Peak Signal-to-Noise Ratio (PSNR) [23], and Structural Similarity Index (SSIM) [18], [19]. These metrics collectively provide an overall performance of the models.

## 3 Results

This section presents the results of reconstructing sparse sampled PAI using the proposed PA OmniNet model and the other models.

**Fig. 3:**
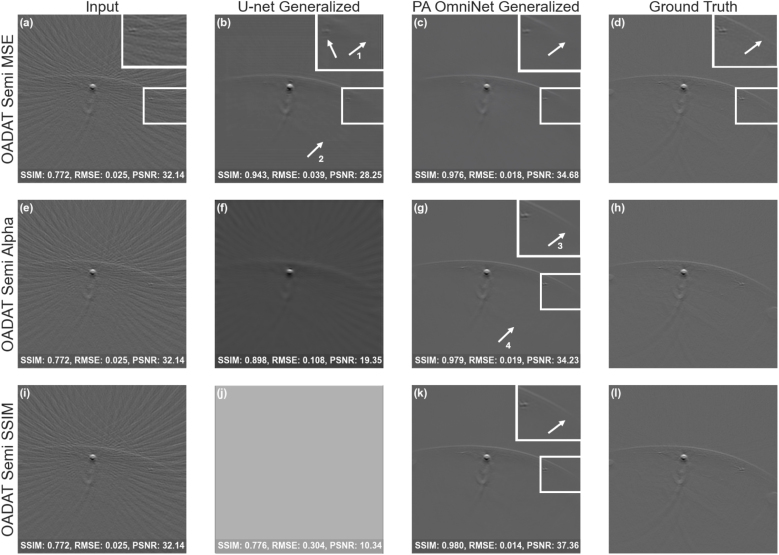
Comparison of U-net Generalized and PA OmniNet Generalized models: (a,e,i) input image, (b,f,j) U-net output, (c,g,k) PA OmniNet output compared to (d,h,l) ground truth for the OADAT dataset for the MSE, Alpha and the SSIM loss functions. .

### 3.1 Performance on OADAT dataset

#### 3.1.1 SWFD semi as the base dataset

We compare PA OmniNet and U-net using the SWFD Semi dataset as the base for generalization. Fig. 3 illustrates the results of the models trained on the three training losses. The best performing U-net model is trained on an MSE loss, as seen in Fig. 3(b) and confirmed by Table 4, with the U-net trained on Alpha delivering a blurry output (Fig. 3(f)) and the U-net trained on SSIM not producing an interpretable output (Fig. 3(j)). The lowest scoring PA OmniNet model is trained on MSE while the Alpha and SSIM trained models deliver higher numerical scores and slightly more detail in the visual output as can be seen in Fig. 3 (g/k). A clear increase in detail (Fig. 3(g) (arrow 3)) and decrease in artifacts (Fig. 3(g) (arrow 4)) can be seen when comparing PA OmniNet Alpha to U-net MSE (Fig. 3 (b/g)). Hereafter, PA OmniNet SWFD Semi trained with the Alpha loss and U-net SWFD Semi trained with the MSE loss will be referred to as PA OmniNet Generalized and U-net Generalized, respectively. For all Specific models, the results of U-net trained on MSE and PA OmniNet trained on Alpha will be shown as these training losses yield the best results for their respected models over all datasets, please refer to the Appendix (6) for all numerical results for all models.

**Table 4:**
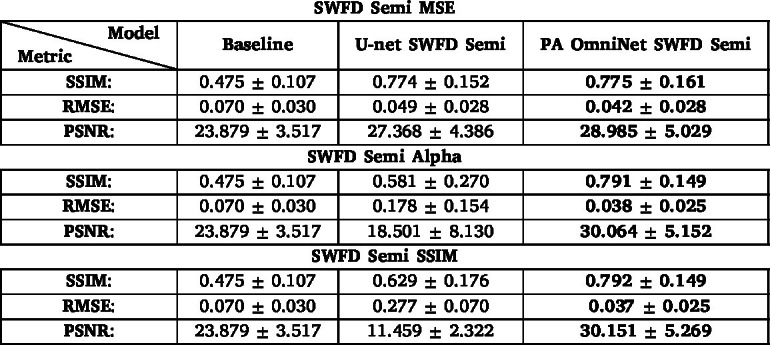
Comparative results obtained for reconstructed images from the SWFD Semi sub-dataset shown in Fig. 3 in terms of figure of metrics: Structural Similarity (SSIM) Index, Root Mean Square Error (RMSE), and Peak Signal-to-Noise Ratio (PSNR) for the MSE, Alpha and the SSIM loss functions. **Bold text** indicates the best possible value for the metric.

#### 3.1.2 Testing on SWFD Multi, MSFD, SCD

**Fig. 4:**
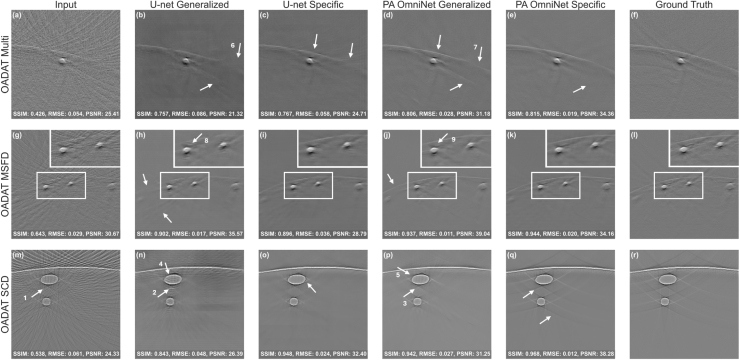
Comparison of models U-net Generalized, U-net Specific, PA OmniNet Generalized and PA OmniNet Specific on test images from SWFD Multi (a - f), MSFD (g - l) and SCD (m - r) respectively.

We evaluated PA OmniNet Generalized by testing it on threedatasets: SWFD Multi, MSFD, and SCD. Additionally, we assessed U-net and PA OmniNet models specifically trained on these datasets. Across these datasets, the PA OmniNet Specific model consistently outperforms the U-net Specific model. Although PA OmniNet Generalized only outperforms U-net Specific once, it outperforms U-net Generalized on every metric, as shown in Table 5. Notably, on the SCD sub-dataset—the most dissimilar to SWFD Semi—PA OmniNet Generalized demonstrates superior generalization capabilities compared to U-net Generalized. PA OmniNet Generalized achieved an SSIM of 0.946, which is marginally lower than the SSIM of the specifically trained U-net (0.957), yet it significantly outperforms the U-net Generalized model, which obtained an SSIM of 0.725, showing its superior generalization capabilities. The Generalized PA OmniNet model removes the deep artifacts (Fig. 4 (arrows 1/2/3)) while maintaining a high resolution subject (Fig. 4(p) (arrow 5)) where U-net Generalized struggles (Fig. 4(n) (arrow 4)). Furthermore, U-net’s output exhibits more artifacts (Fig. 4 (b, h, and n)) compared to the PA OmniNet models in Fig. 4 (d, j and p) while PA OmniNet also outputs finer details which are especially evident in Fig. 4 (d arrow 7) (j arrow 9) compared to Fig. 4 (b arrow 6) (h arrow 8).

**Table 5:**
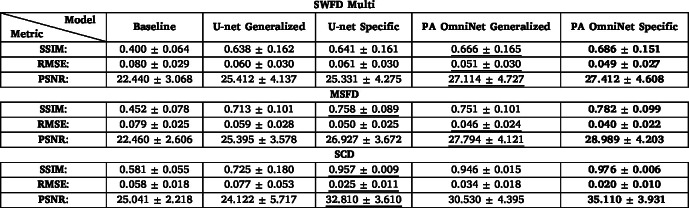
Comparative analysis of results obtained for reconstructed images from the SWFD Multi, MSFD, and SCD sub-datasets using quality metric SSIM, RMSE, and PSNR. **Bold text** indicates the best metric, underlined indicating second best.

#### 3.1.3 Impact of using different context set

**Fig. 5:**
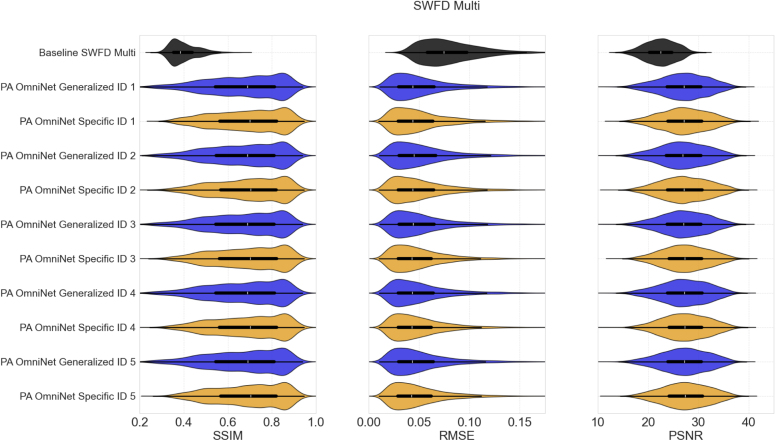
Distribution of SSIM, RMSE and PSNR on the OADAT SWFD Multi sub-dataset using different context sets by changing the patientIDs.

In previous experiments, we used patient 5 for SWFD Semi and Multi and patient 2 for MSFD as context set. Here, we evaluate the impact of varying context set patients on SWFD Multi using both PA OmniNet models. As shown in Fig. 5, altering patients for the context set produces only minor variations in performance. For the SWFD Multi sub-dataset tested on PA OmniNet Generalized, using patient 2 as the context set yielded the highest SSIM (0.6672), whereas patient 3 resulted in the lowest SSIM (0.6659), a difference of only 0.0013. Importantly, even the lowest score remains 0.0283 higher than the U-net Generalized baseline (SSIM: 0.6376). Similarly, for the Specific model, the best performance was achieved with patient 5 (SSIM: 0.6863) and the lowest with patient 3 (SSIM: 0.6827), both outperforming the U-net Specific baseline (SSIM: 0.6410). These results demonstrate that altering the context set within the same application has a minimal impact on PA OmniNet performance, underscoring its robustness.

#### 3.1.4 Impact of context size change

**Fig. 6:**
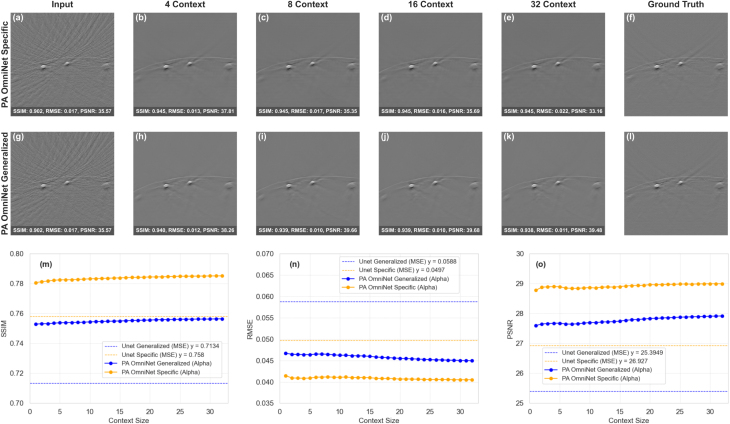
Change in image quality with context size: (a - f) compare PA OmniNet Specific output compared to the input and ground truth, (g - l) compare PA OmniNet Generalized output compared to the input and ground truth, (m, n, and o) shows the SSIM, RMSE, and PSNR change with context size respectively.

Determining the right context set size is crucial to optimize both computational and visual results. We varied the number of image pairs from 1 to 32 to assess its impact on performance. As shown in Fig. 6, both PA OmniNet Specific and Generalized models benefit from increasing context sizes. For both PA OmniNet models, all metrics plateau beyond a context size of 16, indicating that additional pairs provide minimal improvement.

Fig. 6 further illustrates the marginal gains in image quality when increasing the context set size. Both models output visually similar images with minimal difference in fine details.

**Table 6:**
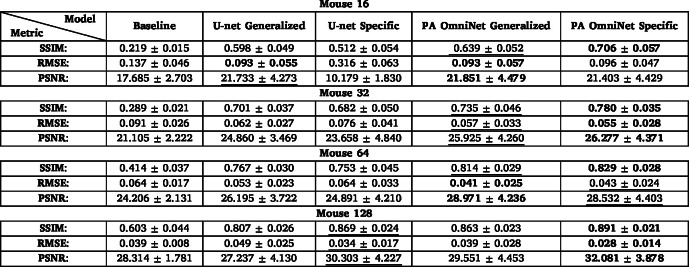
Assessment of reconstructed images from the mouse sub-dataset shown in Fig. 7 in terms of metrics: Structural Similarity (SSIM) Index, Root Mean Square Error (RMSE), and Peak Signal-to-Noise Ratio (PSNR) for 16/32/64/128 sparse sampled configurations. **Bold text** indicates the best possible value for the metric used, underlined indicating second best.

### 3.2 Performance on mouse dataset

#### 3.2.1 In vivo mouse

We evaluated model performance under challenging transducer counts using 16- and 32-sparse, as well as higher 64- and 128-sparse sampling configurations on the mouse sub-datasets. This experiment aims to simulate a broad spectrum of data acquisition scenarios, ranging from challenging, sparsely sampled conditions to more ideal,densely sampled ones. As presented in Table 6, both PA OmniNet models achieved superior SSIM scores across all sampling configurations besides 128. In this specific configuration, the U-net specific (SSIM: 0.869) slightly outperforms PA OmniNet Generalized (SSIM: 0.863) while PA OmniNet Specific still performs best (SSIM: 0.891).

**Fig. 7:**
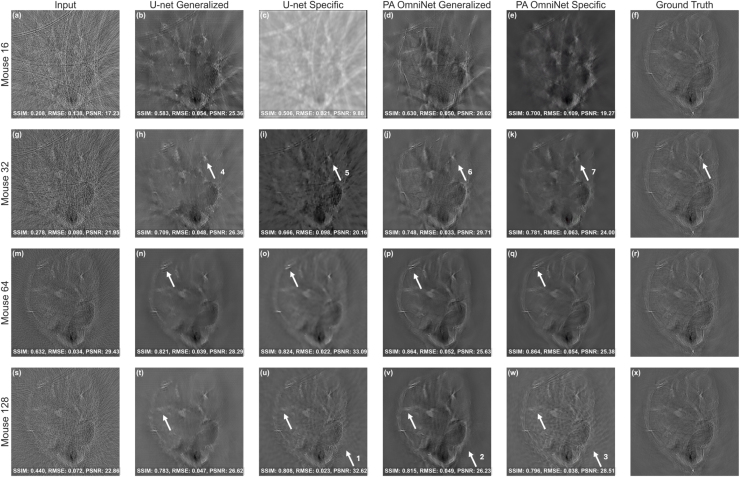
Comparison of models on Mouse dataset: (a, g, m, s) are input images, (b, h, r, x) are U-net Generalized output, (c, i, o, u) are U-net Specific, (d, j, p, v) PA OmniNet Generalized, (e, k, q, w) are PA OmniNet Specific, compared to ground truth in (f, l, r, x), respectively for 16, 32, 64, 128 sparse sampling levels.

Despite this minor difference in SSIM at the highest density, the artifact removal capabilities of the generalized PA OmniNet model were particularly evident. It successfully maintained a smoother background surrounding the region of interest, an area where both dataset-specific models demonstrated limitations (Fig. 7 (u arrow 1) (v arrow 2) (w arrow 3)).

Furthermore, both PA OmniNet models significantly distinguish themselves from the U-net under highly sparse 16- and 32-element sampling conditions. In these scenarios, both PA OmniNet models extracted considerably more detail out of the input images, as can be seen in Fig. 7 (d, e, j, k) and when comparing Fig. 7 (arrows 4, 5, 6, 7), pointing at their effectiveness even in the worst sampling conditions.

#### 3.2.2 Impact of using different application context set

**Fig. 8:**
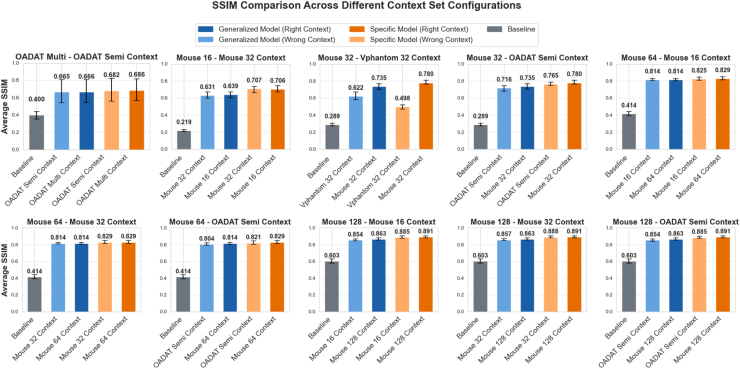
Comparison of average Structural Similarity (SSIM) Index when using different context sets for PA OmniNet Generalized (black) and PA OmniNet Specific (Orange) with lighter shades indicating the usage of the wrong context set and the darker shade indicating the usage of the right context set.

To further evaluate the model’s robustness against variations in the context set, a series of experiments were performed. These tests involved using context sets with lower transducer counts and using context sets derived from different datasets than the input data. As illustrated in Fig. 8, alterations in the context set, while still being an approximate representation, yielded minimal variations in the model’s in output. In the figure, the lighter shade represents tests where the context set originated from a different dataset than the input, while the darker shade indicates tests where both the input and context set were drawn from the same dataset, consistent with earlier experiments. The labels specify the dataset and model configurations tested against mismatched context sets. The model’s robustness is particularly evident in the *Mouse 128 - OADAT Semi Context* scenario, where not only the transducer count is lower than the input but the context originated from a different dataset than the input. Here, the performance of PA OmniNet Generalized had a drop in SSIM from 0.863 to 0.854. PA OmniNet Specific had an even smaller drop, going from an SSIM of 0.891 to 0.885. These marginal drops in visual output underscore the robustness of the model. However, as can be seen in the *Mouse 32 - Vphantom 32 Context*, when the context set is not an approximate representation of the generalization task, the performance drops significantly. PA OmniNet Specific seems to be more sensitive to this, as the drop in performance for PA OmniNet Generalized is less.

#### 3.2.3 Sensitivity analysis

An additional experiment was conducted to evaluate the sensitivity of the proposed model to edges. Transducer counts, ranging from 16 to 64, were employed to assess this sensitivity under both challenging and normal conditions. The sensitivity was quantified by comparing the difference in SSIM between two transducer counts with the corresponding difference in edge similarity between the input and ground truth images of these transducer counts. The sensitivity is calculated using the following formula:

(2) $$Sensitivity=\frac{SSIM\left(N_{j},Gt_{j}\right)-SSIM\left(N_{i},Gt_{i}\right)}{SSIM\left(edge\left(X_{j}\right),Gt_{j}\right)-SSIM\left(edge\left(X_{i}\right),Gt_{i}\right)}$$

Where j and i represent two distinct transducer counts (e.g.,16 and 32, respectively), N being an reconstructed image, X the input, and Gt the Ground truth image. The term $SSIM\left(edge\left(X_{j},Gt_{j}\right)\right)$ denotes the SSIM difference calculated specifically around the edges of the input and ground truth images for the transducer count j. From the results, it is evident that PA OmniNet Specific scores the best with the sensitivity score of 4.07 while PA OmniNet Generalized being second best overall with a sensitivity score of 5.27 as they show minimum sensitivity to the change in the input. Thus, the PA OmniNet Specific and Generalized models are less sensitive compared to the Unet models (Sensitivity score of 5.66 and 9.34 for the U-net Generalized and the U-net Specific models respectively). PA OmniNet Generalized greatly outperforms both the U-net models, underscoring its robustness and superior sensitivity to edges.

**Table 7:**
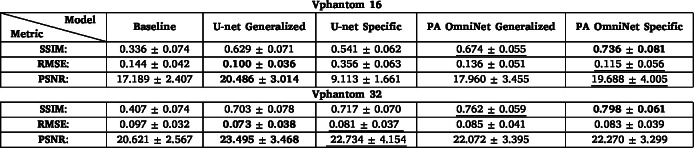
Evaluation of reconstructed images from the Vascular Phantom dataset shown in Fig. 9 in terms of metrics: Structural Similarity (SSIM) Index, Root Mean Square Error (RMSE), and Peak Signal-to-Noise Ratio (PSNR) for 16 and 32 sparse sampled configurations. **Bold text** indicates the best possible value for the metric used, and underlined indicates the second best.

#### 3.2.4 Vascular phantom

**Fig. 9:**
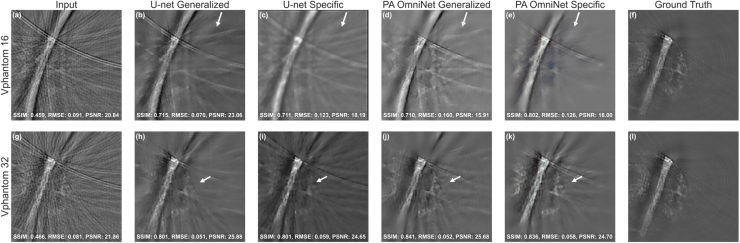
Comparison of models on VPhantom dataset: (a and g) are input images, (b and h) are U-net Generalized output, (c and i) are U-net Specific, (d and j) PA OmniNet Generalized, (e and k) are PA OmniNet Specific, compared to ground truth in (f and l), respectively for 16 and 32 sparse sampling levels.

As shown in Table 7, PA OmniNet models consistently outperform U-net models in both 16 and 32 sparse sampled configurations, with slightly higher results for the Specific PA OmniNet model on both configurations. U-net Specific scores worst on 16 sparse (SSIM: 0.541) as clearly illustrated in Fig. 9(c), with U-net Generalized (SSIM: 0.629) outperforming it, likely due to insufficient training data to train a new U-net model. Both PA OmniNet Generalized (SSIM: 0.674) and Specific (SSIM: 0.736) significantly outperform their U-net counterparts, showing its superior generalization and ability to train on little data. Furthermore, both numerically and visually, PA OmniNet Generalized generalizes better than U-net Generalized, showing more detail and fewer artifacts (Fig. 9 (d, j)).

### 3.3 Overall model performance

To statistically validate and summarize the significance of performance improvements for PA OmniNet, a paired t-test was conducted on the SSIM scores. A comprehensive comparison was performed between PA OmniNet and all relevant scenarios. The proposed Generalized model demonstrates a statistically substantial improvement over U-Net Generalized, yielding a t-score of 3.18 and a p-value of 0.0111. Furthermore, PA OmniNet Generalized exhibits an even more significant improvement when compared to U-Net Specific, achieving a t-score of 2.59 with an associated p-value of 0.032. Additionally, a direct comparison between the two Specific models results in a t-score of 3.364 and a p-value of 0.0065. For a complete overview of all statistical comparisons, please refer to Table 8.

**Fig. 10:**
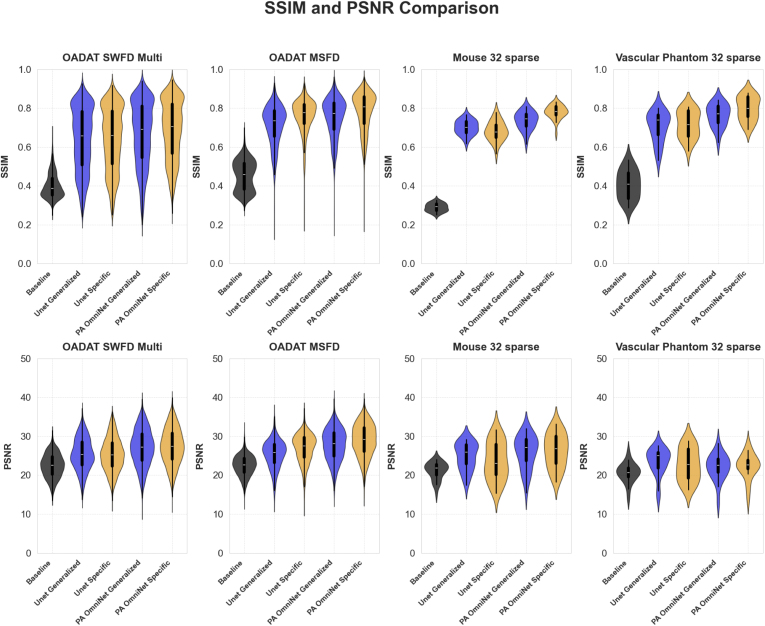
SSIM and PSNR scores across several datasets (SWFD Multi, MSFD, Mouse, Phantom, and Vascular Phantom datasets) and models. PA Omninet Generalized and Specific and U-net Generalized and Specific are compared.

**Table 8:** Performance improvements of PA OmniNet generalized compared to U-net generalized, PA OmniNet specific and U-net specific.

PA OmniNet Generalized (Alpha) Compared to U-Net Generalized (MSE)			

Dataset	SSIM (%)	RMSE (%)	PSNR (dB)

OADAT SWFD Semi	2.26	23.23	2.70
OADAT SWFD Multi	4.47	14.69	1.70
OADAT MSFD	5.28	22.10	2.40
OADAT SCD	30.49	55.99	6.41
Mouse 16 sparse	6.89	0.40	0.12
Mouse 32 sparse	4.96	7.43	1.06
Mouse 64 sparse	6.19	23.92	2.78
Mouse 128 sparse	6.92	20.40	2.31
Vascular Phantom 16 sparse	7.09	−35.50	−2.53
Vascular Phantom 32 sparse	8.41	−16.86	−1.42

Average Improvement	8.30	11.58	1.55

Paired t-test results (PA OmniNet vs. Unet)	t = 3.1846, p = 0.0111	t = 0.8843, p = 0.3996	t = 1.9776, p = 0.0794

PA OmniNet Specific (Alpha) Compared to U-Net Specific (MSE)			

Dataset	SSIM (%)	RMSE (%)	PSNR (dB)

OADAT SWFD Multi	7.06	19.55	2.08
OADAT MSFD	3.13	19.16	2.06
OADAT SCD	1.98	22.06	2.30
Mouse 16 sparse	37.77	69.70	11.22
Mouse 32 sparse	14.36	27.62	2.62
Mouse 64 sparse	10.01	33.06	3.64
Mouse 128 sparse	2.46	19.45	1.78
Vascular Phantom 16 sparse	36.01	67.72	10.58
Vascular Phantom 32 sparse	11.28	−2.53	−0.46

Average Improvement	13.78	30.64	3.98

Paired t-test results (PA OmniNet vs. Unet)	t = 3.6443, p = 0.0065	t = 1.8291, p = 0.1048	t = 2.9319, p = 0.0189
			

PA OmniNet Generalized (Alpha) Compared to U-Net Specific (MSE)			

Dataset	SSIM (%)	RMSE (%)	PSNR (dB)

OADAT SWFD Multi	3.92	15.91	1.78
OADAT MSFD	−0.91	7.75	0.87
OADAT SCD	−1.12	−35.45	−2.28
Mouse 16 sparse	24.64	70.65	11.67
Mouse 32 sparse	7.84	24.39	2.27
Mouse 64 sparse	8.10	36.31	4.08
Mouse 128 sparse	−0.68	−13.26	−0.75
Vascular Phantom 16 sparse	24.53	61.86	8.85
Vascular Phantom 32 sparse	6.32	−5.41	−0.66

Average Improvement	8.07	18.08	2.87

Paired t-test results (PA OmniNet vs. Unet)	t = 2.5917, p = 0.0320	t = 1.6696, p = 0.1336	t = 1.8545, p = 0.1008

## 4 Discussion

**Fig. 11:**
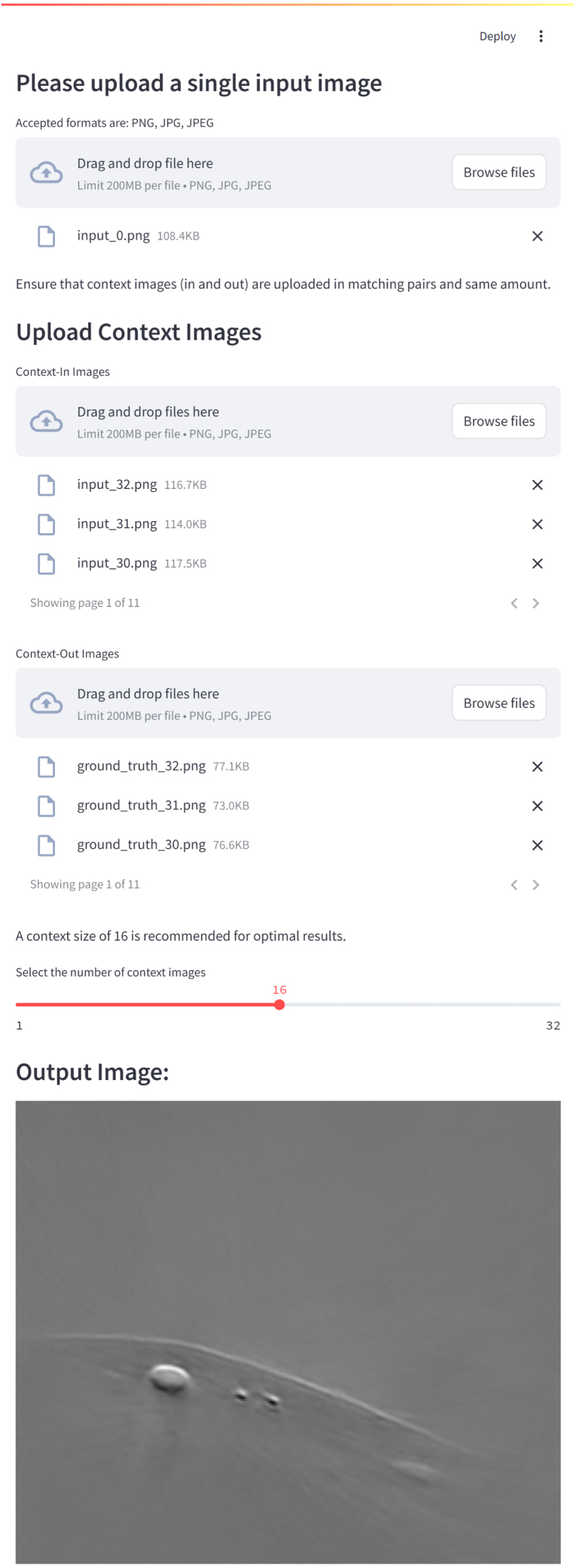
Screenshot of the created app to facilitate easy usage of the model.

We proposed and tested PA OmniNet, a generalizable deep-learning framework that eliminates the need for retraining in order to work across different imaging systems. Our results demonstrate that the PA OmniNet Specific model consistently achieves the highest performance, while the PA OmniNet Generalized model outperforms its U-net counterpart across diverse datasets. Notably, PA OmniNet Generalized surpasses U-net Specific in 66% of our tests while scoring marginally lower on the other 33%, underscoring its strong generalization capabilities without task-specific training (see Table 8 and Fig. 10). This analysis indicates that conditioning deep learning models with a small context set is a promising strategy for enhancing generalization in photoacoustic imaging (PAI). Beyond its robust generalization in a single forward pass, PA OmniNet is particularly well suited for scenarios with limited data availability, demonstrated by its performance on the vascular phantom sub-dataset (Section 3.2.1). While U-net Generalized performs adequately on datasets similar to the OADAT SWFD Semi, PA OmniNet Generalized demonstrates superior adaptability on more dissimilar datasets. This highlights a key advantage of the PA OmniNet architecture over traditional U-net models in addressing the common challenge of data scarcity in photoacoustic imaging. A further critical finding concerns the optimal context size during training and testing. Our tests reveal that models trained with larger context sizes tend to under perform when presented with smaller context sets, whereas models trained with smaller context sizes can effectively leverage larger context sizes to a certain point. This suggests that an overly large training context may induce overdependence on the provided context, thereby reducing flexibility. Based on both qualitative assessments, we identify an optimal training context size of 4 and a testing context size of 16. After various comparisons between models, metrics and visual output, PA OmniNet models trained with the Alpha loss are seen to be the most robust and best performing ones. While U-net models often fail to perform well when trained with this loss, the robustness of PA OmniNet allows the model to be trained with an eye on visual output by combining SSIM and MSE in the training loss. The robustness of the model is further supported by the excellent results when using sub-optimal context sets. When these sets differ in transducer count, acquisition angle, or application, minimal degradation is seen in the output, promoting the model’s practical application. The PA OmniNet framework presented in this study is inspired by the Neuralizer architecture, originally proposed for multi-task learning in MRI images, capable of generalizing across various imaging modalities without retraining. In our current implementation, PA OmniNet has successfully demonstrated robust generalization for reconstruction-related tasks within the scope of openly available datasets in PAI, such as sparse sampling, limited-view reconstruction, and differences in transducer geometry. Given the generalizable architecture of PA OmniNet, the next step would involve extending it explicitly into a multi-task learning in PAI. Specifically, the network could be simultaneously trained to address multiple tasks beyond reconstruction, including image segmentation, artifact removal, and chromophore quantification. Exploring datasets acquired from different imaging systems, such as varying frequency bands, transducer geometries, and illumination schemes, would provide valuable insights into the model’s robustness. To explore these aspects,an open-source application is made available to the community https://github.com/olivierstam4/PA_OmniNet (Fig. 11). Integrating multi-task capability into PA OmniNet could offer a step toward the development of universal PAI models. Further future work could integrate the context set approach into other high-performance deep learning architectures and extend its application to tasks such as denoising and segmentation. Such advancements could lower the threshold in the development of photoacoustic systems, by reducing both cost and expertise requirements, and ultimately accelerating clinical translation. To support these efforts, we have made our GitHub repository publicly available and developed an application that allows users to upload context images to test new datasets.

## 5 Conclusion

In this work, we introduced PA OmniNet, a deep learning model that leverages a small context set to improve generalization across different photoacoustic imaging scenarios without retraining. We demonstrate that PA OmniNet Generalized achieves superior performance relative to specifically trained U-net models on a new dataset, eliminating the need for task-specific retraining and lowering computational overhead. The development of a user-friendly interface that allows dynamic adjustment of the context size further supports its practical application to the community. Nonetheless, our study has limitations. The model was evaluated on a limited number of datasets. Additionally, while PA OmniNet shows promise in generalization, further research is necessary to enhance its robustness and applicability to a broader range of tasks and applications. PA OmniNet represents an initial step toward developing generalized deep-learning frameworks for photoacoustic imaging. Future work will focus on addressing these limitations and applying them to application-specific tasks to find utility in clinical settings.

## CRediT authorship contribution statement

**Olivier J.M. Stam:** Writing – original draft, Visualization, Validation, Formal analysis, Data curation. **Kalloor Joseph Francis:** Writing – review & editing, Writing – original draft, Supervision, Methodology, Conceptualization. **Navchetan Awasthi:** Writing – review & editing, Writing – original draft, Visualization, Validation, Supervision, Methodology, Investigation, Conceptualization.

## Declaration of competing interest

The authors declare that they have no known competing financial interests or personal relationships that could have appeared to influence the work reported in this paper.

## Data Availability

I have shared the code repository for all the codes used in this work.

## Appendix A Supplementary data

MMC S1 Additional numerical data showing model performance on various datasets using different training losses.
