# Supplementary material for: PA OmniNet: A retraining-free, generalizable deep learning framework for robust photoacoustic image reconstruction
Source: Photoacoustics. 2025 Jul 8;45:100740. doi: 10.1016/j.pacs.2025.100740 (PMC12284555; doi:10.1016/j.pacs.2025.100740)
Supplement: MMC S1 — Additional numerical data showing model performance on various datasets using different training losses. [file mmc1.pdf]

Table 9
Comparative analysis of results obtained for reconstructed images from the SWFD Semi dataset using quality metric SSIM, RMSE, and PSNR. Results are shown for all models on all different training losses, the U-net MSE and PA OmniNet Alpha models are used for comparisons in this paper. **Bold text** indicates the best metric.

Metric \ Model	Baseline	U-net SWFD Semi	PA OmniNet SWFD Semi
**SWFD Semi MSE**			
**SSIM**:	0.475 ± 0.107	0.774 ± 0.152	**0.775** ± **0.161**
**RMSE**:	0.070 ± 0.030	0.049 ± 0.028	**0.042** ± **0.028**
**PSNR**:	23.879 ± 3.517	27.368 ± 4.386	**28.985** ± **5.029**
**SWFD Semi Alpha**			
**SSIM**:	0.475 ± 0.107	0.581 ± 0.270	**0.791** ± **0.149**
**RMSE**:	0.070 ± 0.030	0.178 ± 0.154	**0.038** ± **0.025**
**PSNR**:	23.879 ± 3.517	18.501 ± 8.130	30.064 ± 5.152
**SWFD Semi SSIM**			
**SSIM**:	0.475 ± 0.107	0.629 ± 0.176	**0.792** ± **0.149**
**RMSE**:	0.070 ± 0.030	0.277 ± 0.070	**0.037** ± **0.025**
**PSNR**:	23.879 ± 3.517	11.459 ± 2.322	**30.151** ± **5.269**

Table 10
Comparative analysis of results obtained for reconstructed images from the SWFD Multi dataset using quality metric SSIM, RMSE, and PSNR. Results are shown for all models on all different training losses, the U-net MSE and PA OmniNet Alpha models are used for comparisons in this paper. **Bold text** indicates the best metric, <u>underlined text</u> second best.

Metric \ Model	Baseline	U-net Generalized	U-net Specific	PA OmniNet Generalized	PA OmniNet Specific
**Multi MSE**					
**SSIM**:	0.400 ± 0.064	0.638 ± 0.162	0.641 ± 0.161	<u>0.647 ± 0.178</u>	**0.660** ± **0.173**
**RMSE**:	0.080 ± 0.029	0.060 ± 0.030	0.061 ± 0.030	<u>0.053 ± 0.030</u>	**0.049** ± **0.028**
**PSNR**:	22.440 ± 3.068	25.412 ± 4.137	25.331 ± 4.275	<u>26.660 ± 4.509</u>	**27.474** ± **4.612**
**Multi Alpha**					
**SSIM**:	0.400 ± 0.064	0.439 ± 0.266	0.410 ± 0.158	<u>0.666 ± 0.165</u>	**0.686** ± **0.151**
**RMSE**:	0.080 ± 0.029	0.210 ± 0.165	0.566 ± 0.063	<u>0.051 ± 0.030</u>	**0.049** ± **0.027**
**PSNR**:	22.440 ± 3.068	17.052 ± 8.369	4.996 ± 0.977	<u>27.114 ± 4.727</u>	**27.412** ± **4.608**
**Multi SSIM**					
**SSIM**:	0.400 ± 0.064	0.505 ± 0.190	0.448 ± 0.171	<u>0.669 ± 0.164</u>	**0.688** ± **0.152**
**RMSE**:	0.080 ± 0.029	0.266 ± 0.062	0.440 ± 0.063	<u>0.051 ± 0.029</u>	**0.048** ± **0.027**
**PSNR**:	22.440 ± 3.068	11.759 ± 2.156	7.219 ± 1.263	<u>27.152 ± 4.679</u>	**27.522** ± **4.613**

Table 11
Comparative analysis of results obtained for reconstructed images from the SCD dataset using quality metric SSIM, RMSE, and PSNR. Results are shown for all models on all different training losses, the U-net MSE and PA OmniNet Alpha models are used for comparisons in this paper. **Bold text** indicates the best metric, underlined text second best.

Metric \ Model	Baseline	U-net Generalized	U-net Specific	PA OmniNet Generalized	PA OmniNet Specific
**SCD MSE**					
**SSIM**:	0.581 ± 0.055	0.725 ± 0.180	0.957 ± 0.009	0.934 ± 0.014	**0.963 ± 0.009**
**RMSE**:	0.058 ± 0.018	0.077 ± 0.053	0.025 ± 0.011	0.034 ± 0.013	**0.024 ± 0.012**
**PSNR**:	25.041 ± 2.218	24.122 ± 5.717	32.810 ± 3.610	29.857 ± 2.843	**33.255 ± 3.821**
**SCD Alpha**					
**SSIM**:	0.581 ± 0.055	0.747 ± 0.250	0.863 ± 0.154	0.946 ± 0.015	**0.976 ± 0.006**
**RMSE**:	0.058 ± 0.018	0.168 ± 0.135	0.077 ± 0.071	0.034 ± 0.018	**0.020 ± 0.010**
**PSNR**:	25.041 ± 2.218	18.141 ± 6.862	24.396 ± 5.562	30.530 ± 4.395	**35.110 ± 3.931**
**SCD SSIM**					
**SSIM**:	0.581 ± 0.055	0.751 ± 0.077	0.669 ± 0.085	0.946 ± 0.015	**0.977 ± 0.006**
**RMSE**:	0.058 ± 0.018	0.300 ± 0.077	0.455 ± 0.077	0.027 ± 0.013	**0.020 ± 0.010**
**PSNR**:	25.041 ± 2.218	10.764 ± 2.348	6.967 ± 1.512	32.069 ± 3.683	**34.895 ± 3.968**

Table 12
Comparative analysis of results obtained for reconstructed images from the MSFD dataset using quality metric SSIM, RMSE, and PSNR. Results are shown for all models on all different training losses, the U-net MSE and PA OmniNet Alpha models are used for comparisons in this paper. **Bold text** indicates the best metric, underlined text second best.

Metric \ Model	Baseline	U-net Generalized	U-net Specific	PA OmniNet Generalized	PA OmniNet Specific
**MSFD MSE**					
**SSIM**:	0.452 ± 0.078	0.713 ± 0.101	**0.758 ± 0.089**	0.740 ± 0.107	0.755 ± 0.103
**RMSE**:	0.079 ± 0.025	0.059 ± 0.028	0.050 ± 0.025	0.048 ± 0.024	**0.042 ± 0.023**
**PSNR**:	22.460 ± 2.606	25.395 ± 3.578	26.927 ± 3.672	27.277 ± 3.789	**28.592 ± 4.225**
**MSFD Alpha**					
**SSIM**:	0.452 ± 0.078	0.612 ± 0.215	0.452 ± 0.092	0.751 ± 0.101	**0.782 ± 0.099**
**RMSE**:	0.079 ± 0.025	0.141 ± 0.126	0.608 ± 0.069	0.046 ± 0.024	**0.040 ± 0.022**
**PSNR**:	22.460 ± 2.606	20.023 ± 7.121	4.378 ± 1.020	27.794 ± 4.121	**28.989 ± 4.203**
**MSFD SSIM**					
**SSIM**:	0.452 ± 0.078	0.571 ± 0.108	0.577 ± 0.109	0.752 ± 0.100	**0.782 ± 0.100**
**RMSE**:	0.079 ± 0.025	0.307 ± 0.069	0.293 ± 0.069	0.045 ± 0.023	**0.040 ± 0.021**
**PSNR**:	22.460 ± 2.606	10.495 ± 2.149	10.939 ± 2.274	27.903 ± 4.029	**29.070 ± 4.163**

Table 13
Comparative analysis of results obtained for reconstructed images from the Mouse dataset on a 16 transducer acquisition setup using quality metric SSIM, RMSE, and PSNR. Results are shown for all models on all different training losses, the U-net MSE and PA OmniNet Alpha models are used for comparisons in this paper. **Bold text** indicates the best metric, underlined text second best.

Mouse 16 MSE

Model / Metric	Baseline	U-net Generalized	U-net Specific	PA OmniNet Generalized	PA OmniNet Specific
**SSIM**:	0.219 ± 0.015	**0.598 ± 0.049**	0.512 ± 0.054	0.444 ± 0.120	0.481 ± 0.043
**RMSE**:	0.137 ± 0.046	**0.093 ± 0.055**	0.316 ± 0.063	0.107 ± 0.046	0.120 ± 0.037
**PSNR**:	17.685 ± 2.703	**21.733 ± 4.273**	10.179 ± 1.830	20.154 ± 3.607	18.761 ± 2.580

Mouse 16 Alpha

**SSIM**:	0.219 ± 0.015	0.533 ± 0.187	0.696 ± 0.037	0.639 ± 0.052	**0.706 ± 0.057**
**RMSE**:	0.137 ± 0.046	0.169 ± 0.111	0.114 ± 0.059	**0.093 ± 0.057**	0.096 ± 0.047
**PSNR**:	17.685 ± 2.703	17.742 ± 6.930	19.820 ± 3.967	**21.851 ± 4.479**	21.403 ± 4.429

Mouse 16 SSIM

**SSIM**:	0.219 ± 0.015	0.559 ± 0.060	**0.661 ± 0.052**	0.639 ± 0.050	0.634 ± 0.059
**RMSE**:	0.137 ± 0.046	0.339 ± 0.055	0.207 ± 0.063	**0.088 ± 0.051**	0.119 ± 0.053
**PSNR**:	17.685 ± 2.703	9.501 ± 1.474	14.118 ± 2.935	**22.185 ± 4.319**	19.330 ± 3.844

Table 14
Comparative analysis of results obtained for reconstructed images from the Mouse dataset on a 32 transducer acquisition setup using quality metric SSIM, RMSE, and PSNR. Results are shown for all models on all different training losses, the U-net MSE and PA OmniNet Alpha models are used for comparisons in this paper. **Bold text** indicates the best metric, underlined text second best.

Mouse 32 MSE

Model / Metric	Baseline	U-net Generalized	U-net Specific	PA OmniNet Generalized	PA OmniNet Specific
**SSIM**:	0.289 ± 0.021	**0.701 ± 0.037**	0.682 ± 0.050	**0.701 ± 0.053**	0.651 ± 0.063
**RMSE**:	0.091 ± 0.026	**0.062 ± 0.027**	0.076 ± 0.041	0.085 ± 0.042	0.106 ± 0.052
**PSNR**:	21.105 ± 2.222	**24.860 ± 3.469**	23.658 ± 4.840	22.231 ± 3.728	20.680 ± 4.773

Mouse 32 Alpha

**SSIM**:	0.289 ± 0.021	0.676 ± 0.058	0.772 ± 0.037	0.735 ± 0.046	**0.780 ± 0.035**
**RMSE**:	0.091 ± 0.026	0.071 ± 0.040	0.058 ± 0.030	0.057 ± 0.033	**0.055 ± 0.028**
**PSNR**:	21.105 ± 2.222	24.177 ± 4.642	25.592 ± 3.803	25.925 ± 4.260	**26.277 ± 4.371**

Mouse 32 SSIM

**SSIM**:	0.289 ± 0.021	0.559 ± 0.060	0.768 ± 0.030	0.738 ± 0.043	**0.779 ± 0.033**
**RMSE**:	0.091 ± 0.026	0.339 ± 0.055	0.062 ± 0.024	**0.053 ± 0.029**	0.057 ± 0.030
**PSNR**:	21.105 ± 2.222	9.501 ± 1.474	24.776 ± 3.222	**26.266 ± 3.564**	25.866 ± 4.106

Table 15
Comparative analysis of results obtained for reconstructed images from the Mouse dataset on a 64 transducer acquisition setup using quality metric SSIM, RMSE, and PSNR. Results are shown for all models on all different training losses, the U-net MSE and PA OmniNet Alpha models are used for comparisons in this paper. **Bold text** indicates the best metric, <u>underlined text</u> second best.

Mouse 64 MSE

Metric \ Model	Baseline	U-net Generalized	U-net Specific	PA OmniNet Generalized	PA OmniNet Specific
**SSIM**:	0.414 ± 0.037	<u>0.767 ± 0.030</u>	0.753 ± 0.045	**0.798 ± 0.032**	0.624 ± 0.058
**RMSE**:	0.064 ± 0.017	<u>0.053 ± 0.023</u>	0.064 ± 0.033	**0.050 ± 0.021**	0.093 ± 0.046
**PSNR**:	24.206 ± 2.131	<u>26.195 ± 3.722</u>	24.891 ± 4.210	**26.759 ± 3.547**	21.685 ± 4.486

Mouse 64 Alpha

Metric \ Model	Baseline	U-net Generalized	U-net Specific	PA OmniNet Generalized	PA OmniNet Specific
**SSIM**:	0.414 ± 0.037	0.710 ± 0.082	0.812 ± 0.031	<u>0.814 ± 0.029</u>	**0.829 ± 0.028**
**RMSE**:	0.064 ± 0.017	0.086 ± 0.068	0.061 ± 0.026	**0.041 ± 0.025**	<u>0.043 ± 0.024</u>
**PSNR**:	24.206 ± 2.131	23.048 ± 5.272	24.986 ± 3.648	**28.971 ± 4.236**	<u>28.532 ± 4.403</u>

Mouse 64 SSIM

Metric \ Model	Baseline	U-net Generalized	U-net Specific	PA OmniNet Generalized	PA OmniNet Specific
**SSIM**:	0.414 ± 0.037	0.559 ± 0.060	0.807 ± 0.031	<u>0.814 ± 0.031</u>	**0.830 ± 0.028**
**RMSE**:	0.064 ± 0.017	0.339 ± 0.055	0.069 ± 0.031	<u>0.040 ± 0.022</u>	**0.039 ± 0.023**
**PSNR**:	24.206 ± 2.131	9.501 ± 1.474	24.095 ± 3.873	<u>28.940 ± 4.234</u>	**29.367 ± 4.331**

Table 16
Comparative analysis of results obtained for reconstructed images from the Mouse dataset on a 128 transducer acquisition setup using quality metric SSIM, RMSE, and PSNR. Results are shown for all models on all different training losses, the U-net MSE and PA OmniNet Alpha models are used for comparisons in this paper. **Bold text** indicates the best metric, <u>underlined text</u> second best.

Mouse 128 MSE

Metric \ Model	Baseline	U-net Generalized	U-net Specific	PA OmniNet Generalized	PA OmniNet Specific
**SSIM**:	0.603 ± 0.044	0.807 ± 0.026	**0.869 ± 0.024**	0.844 ± 0.025	<u>0.865 ± 0.025</u>
**RMSE**:	0.039 ± 0.008	0.049 ± 0.025	**0.034 ± 0.017**	<u>0.037 ± 0.021</u>	0.039 ± 0.022
**PSNR**:	28.314 ± 1.781	27.237 ± 4.130	**30.303 ± 4.227**	<u>29.650 ± 4.231</u>	29.503 ± 4.636

Mouse 128 Alpha

Metric \ Model	Baseline	U-net Generalized	U-net Specific	PA OmniNet Generalized	PA OmniNet Specific
**SSIM**:	0.603 ± 0.044	0.727 ± 0.121	0.474 ± 0.061	<u>0.863 ± 0.023</u>	**0.891 ± 0.021**
**RMSE**:	0.039 ± 0.008	0.091 ± 0.081	0.588 ± 0.055	<u>0.039 ± 0.028</u>	**0.028 ± 0.014**
**PSNR**:	28.314 ± 1.781	22.929 ± 5.749	4.657 ± 0.828	<u>29.551 ± 4.453</u>	**32.081 ± 3.878**

Mouse 128 SSIM

Metric \ Model	Baseline	U-net Generalized	U-net Specific	PA OmniNet Generalized	PA OmniNet Specific
**SSIM**:	0.603 ± 0.044	0.559 ± 0.060	<u>0.875 ± 0.024</u>	0.866 ± 0.022	**0.891 ± 0.019**
**RMSE**:	0.039 ± 0.008	0.339 ± 0.055	0.041 ± 0.022	<u>0.034 ± 0.015</u>	**0.024 ± 0.010**
**PSNR**:	28.314 ± 1.781	9.501 ± 1.474	28.742 ± 4.374	<u>30.182 ± 3.587</u>	**32.843 ± 3.249**

Table 17
Comparative analysis of results obtained for reconstructed images from the Vphantom dataset on a 16 transducer acquisition setup using quality metric SSIM, RMSE, and PSNR. Results are shown for all models on all different training losses, the U-net MSE and PA OmniNet Alpha models are used for comparisons in this paper. **Bold text** indicates the best metric, underlined text second best.

Vphantom 16 MSE

Model / Metric	Baseline	U-net Generalized	U-net Specific	PA OmniNet Generalized	PA OmniNet Specific
**SSIM**:	0.336 ± 0.074	0.629 ± 0.071	0.541 ± 0.062	**0.655 ± 0.101**	0.632 ± 0.104
**RMSE**:	0.144 ± 0.042	0.100 ± 0.036	0.356 ± 0.063	**0.118 ± 0.048**	0.154 ± 0.065
**PSNR**:	17.189 ± 2.407	**20.486 ± 3.014**	9.113 ± 1.661	19.246 ± 3.575	16.894 ± 3.394

Vphantom 16 Alpha

**SSIM**:	0.336 ± 0.074	0.524 ± 0.238	**0.748 ± 0.061**	0.674 ± 0.055	0.736 ± 0.081
**RMSE**:	0.144 ± 0.042	0.194 ± 0.112	**0.110 ± 0.044**	0.136 ± 0.051	0.115 ± 0.056
**PSNR**:	17.189 ± 2.407	15.762 ± 5.475	19.750 ± 3.309	17.960 ± 3.455	**19.688 ± 4.005**

Vphantom 16 SSIM

**SSIM**:	0.336 ± 0.074	0.572 ± 0.057	**0.758 ± 0.075**	0.654 ± 0.063	0.756 ± 0.070
**RMSE**:	0.144 ± 0.042	0.343 ± 0.065	**0.092 ± 0.047**	0.146 ± 0.050	0.099 ± 0.048
**PSNR**:	17.189 ± 2.407	9.469 ± 1.848	**21.561 ± 3.743**	17.255 ± 3.217	20.692 ± 3.140

Table 18
Comparative analysis of results obtained for reconstructed images from the Vphantom dataset on a 32 transducer acquisition setup using quality metric SSIM, RMSE, and PSNR. Results are shown for all models on all different training losses, the U-net MSE and PA OmniNet Alpha models are used for comparisons in this paper. **Bold text** indicates the best metric, underlined text second best.

Vphantom 32 MSE

Model / Metric	Baseline	U-net Generalized	U-net Specific	PA OmniNet Generalized	PA OmniNet Specific
**SSIM**:	0.407 ± 0.074	0.703 ± 0.078	0.717 ± 0.070	**0.760 ± 0.081**	0.756 ± 0.088
**RMSE**:	0.097 ± 0.032	**0.073 ± 0.038**	0.081 ± 0.037	0.078 ± 0.040	0.089 ± 0.044
**PSNR**:	20.621 ± 2.567	**23.495 ± 3.468**	22.734 ± 4.154	23.040 ± 3.877	22.007 ± 4.348

Vphantom 32 Alpha

**SSIM**:	0.407 ± 0.074	0.628 ± 0.179	0.670 ± 0.073	0.762 ± 0.059	**0.798 ± 0.061**
**RMSE**:	0.097 ± 0.032	0.137 ± 0.117	0.268 ± 0.062	0.085 ± 0.041	**0.083 ± 0.039**
**PSNR**:	20.621 ± 2.567	19.588 ± 6.156	11.675 ± 2.091	22.072 ± 3.395	**22.270 ± 3.299**

Vphantom 32 SSIM

**SSIM**:	0.407 ± 0.074	0.572 ± 0.057	0.801 ± 0.058	0.741 ± 0.074	**0.802 ± 0.060**
**RMSE**:	0.097 ± 0.032	0.343 ± 0.065	**0.074 ± 0.034**	0.105 ± 0.052	0.079 ± 0.037
**PSNR**:	20.621 ± 2.567	9.469 ± 1.848	**23.300 ± 3.225**	20.541 ± 4.316	22.694 ± 3.353
